# Selection of Reference Genes for Quantitative Gene Expression Studies in *Platycladus orientalis* (Cupressaceae) Using Real-Time PCR

**DOI:** 10.1371/journal.pone.0033278

**Published:** 2012-03-30

**Authors:** Ermei Chang, Shengqing Shi, Jianfeng Liu, Tielong Cheng, Liang Xue, Xiuyan Yang, Wenjuan Yang, Qian Lan, Zeping Jiang

**Affiliations:** 1 State Key Laboratory of Tree Genetics and Breeding, Research Institute of Forestry, Chinese Academy of Forestry, Beijing, People’s Republic of China; 2 Sci-tech Management Division, Chinese Academy of Forestry, Beijing, People’s Republic of China; Korea University, Republic of Korea

## Abstract

*Platycladus orientalis* is a tree species that is highly resistant, widely adaptable, and long-lived, with lifespans of even thousands of years. To explore the mechanisms underlying these characteristics, gene expressions have been investigated at the transcriptome level by RNA-seq combined with a digital gene expression (DGE) technique. So, it is crucial to have a reliable set of reference genes to normalize the expressions of genes in *P. orientalis* under various conditions using the most accurate and sensitive method of quantitative real-time PCR (qRT-PCR). In this study, we selected 10 reference gene candidates from transcriptome data of *P. orientalis*, and examined their expression profiles by qRT-PCR using 29 different samples of *P. orientalis*, which were collected from plants of different ages, different tissues, and plants subjected to different treatments including cold, heat, salinity, polyethylene glycol (PEG), and abscisic acid (ABA). Three analytical software packages (geNorm, Bestkeeper, and NormFinder) were used to assess the stability of gene expression. The results showed that ubiquitin-conjugating enzyme E2 (*UBC*) and alpha-tubulin (*aTUB*) were the optimum pair of reference genes at all developmental stages and under all stress conditions. *ACT7* was the most stable gene across different tissues and cold-treated samples, while *UBQ* was the most stably expressed reference gene for NaCl- and ABA-treated samples. In parallel, *aTUB* and *UBC* were used singly or in combination as reference genes to examine the expression levels of *NAC* (a homolog of *AtNAC2*) in plants subjected to various treatments with qRT-PCR. The results further proved the reliability of the two selected reference genes. Our study will benefit future research on the expression of genes in response to stress/senescence in *P. orientalis* and other members of the Cupressaceae.

## Introduction

Quantitative real-time PCR (qRT-PCR) allows sensitive, specific, and reproducible quantification of nucleic acids [1], and it has been widely used to analyze mRNA in different organisms, transgenic and gene mutation experiments, and to identify parasitic organisms [2]–[5]. However, there are substantial variations in RNA stability, quantity, purity, and the efficiency of reverse transcription (RT) and polymerase chain reactions (PCR) [6]. Therefore, it is important to select a suitable reference gene and to use a set of standardized experimental conditions to accurately quantify gene expression by qRT-PCR; otherwise, it may give inaccurate results [7]–[14].

**Table 1:** Descriptions of candidate genes from *Platycladus orientalis* for qRT-PCR.

Gene symbol	Gene name	*Arabidopsis* homolog locus	Primer sequence (5'–3')	Size (bp)	PCR efficiency
GAPDH	Glyceraldehyde-3-Phosphate dehydrogenase	AT1G79530	GAGATTCCATGGGGTGATTTTG	150	99.2%
			AACCACAAACATAGGGGCATC		
ACT7	Actin 7	AT5G09810	GGAGGTTCCACCATGTTTCC	152	101.1%
			GTGCTGAGCGAAGCGAGAAT		
aTUB	Alpha-tubulin	AT5G19770	CCACATCTCTTAGGTTTGATGGAG	205	103.6%
			GGGTCACACTTGGCCATCAT		
bTUB	Beta tubulin	AT1G20010	TCCCATCGCCTAAGGTATCG	197	101.6%
			TCCGCTCATTGTCGCAGATA		
UBC	Ubiquitin-conjugating enzyme E2	AT3G57870	TCTTGCTGAAGAGCGGAAGG	108	99.2%
			ATGCACTGCCAGACCATCAA		
UBQ	Ubiquitin 10	AT5G20620	AGGGGAGGCATGCAGATTTT	120	101.9%
			AGGAATGCCCTCCTTGTCCT		
EF1a	Elongation factor 1-alpha	AT1G07940	TCTGCCCCTTCAGGATGTTT	144	98%
			TGCATCTCGACGGACTTGAC		
DNAJ	DanJ-like protein	AT3G44110	TTCGTGAAGGCACACAGCAT	133	98.9%
			TGCCTGTGTGTCAGGTTCGT		
SAND	Sand family protein	AT2G28390	TGGTGGTCTGCATGTGGAAG	134	95.5%
			CCGGACCTCCAATTCCAATA		
CAC	Clathrin adaptor complexes	AT1G56590	ACTGGGGAAGTAATGCTTGAGA	100	104.2%
			AAGAATCTCTCACGGGAAAAGC		
NAC	NAC domain protein	AT5G39610	AGAGGAGAAGGAAGCGAAGG	169	104.3%
			TGGCGTATGATGAGTCCAAA		

Note: All reference gene sequences from transcriptome data of *Platycladus orientalis* were searched with BLAST using sequences of *Arabidopsis thaliana* in GenBank. Sequences of candidate housekeeping genes and NAC domain protein gene are provided in the Supporting Information.

Reference genes are those that are constitutively expressed and are required for cellular survival. They include genes that encode products with functions in maintaining cell wall structure and primary metabolism. Some examples of reference genes include 18S ribosomal RNA (*18S rRNA*), actin (*ACT*), tubulin (*TUB*), glyceraldehyde-3-phosphate dehydrogenase (*GAPDH*), polyubiquitin (*UBQ*), and elongation factor 1-a (*EF1a*) [15], [16], [17]. Some of them, such as *EF1a*, *ACT2*, and *TUA*, do not satisfy certain basic requirements for use as an internal control in *Arabidopsis thaliana* [16] and tomato [17]. Recently, some novel reference genes that show highly stable expression were identified from analyses of microarray data sets from *A. thaliana*. These new reference genes include *SAND* and *TIP41*, which encode a SAND family protein and a TIP41-like family protein, respectively [16]. Both of these reference genes outperform the classical ones; for example, *CAC* and *TIP41* were also among the most stably expressed genes in tomato. A recent study demonstrated that even some stress-related genes can serve as reference genes in some experiments, such as those encoding *SKP1*/Ask-interacting protein 16 (*SKIP16*), metalloprotease (*MTP*), RNA polymerase subunit (*RPII*), and F-box protein (*F-box*) [18]–[22]. Therefore, it is important to select a suitable reference gene with a constant level of expression under certain experimental conditions and among various species [16].

To date, many stable reference genes have been screened in both model and crop species, such as *A. thaliana* [23], rice [24], [25], *Brachypodium distachyon* [26], wheat [27], barley [28], soybean [29], [30], tomato [31], potato [32], sugarcane [33] and poplar [34]. However, no suitable internal controls for gene expression studies have been defined for *Platycladus orientalis*, which limits further studies on this species at the transcriptome level.

*P*. *orientalis*, as a member of the Cupressaceae, is used extensively as a medicinal ingredient and as an ornamental landscape plant that can tolerate a wide range of environmental extremes [35], [36]. Many individuals of this species have lifespans of one to several thousands of years in China [37]. However, why do some trees, such as *P*. *orientalis*, live for so long? This question has received much attention in recent years [38], [39]. The long lifespan and hardiness of *P*. *orientalis* make it an ideal material in which to study expression patterns of genes related to stress responses and longevity of tree species. In our previous research, the transcriptome and digital expression profiles were compared between young and old trees of *P. orientalis* by RNA-seq combined with the digital gene expression (DGE) technique (data not shown). To further elucidate the excellent genetic traits of *P. orientalis*, further research is required to analyze expression of particular genes at various developmental stages and under certain stress conditions. However, several studies have shown that the transcription levels of genes vary considerably under various experimental conditions [40], [41], [42]. Therefore, it is urgent to identify a set of stable reference genes for further analyses of gene expression profiles in *P*. *orientalis*.

**Figure 1:**
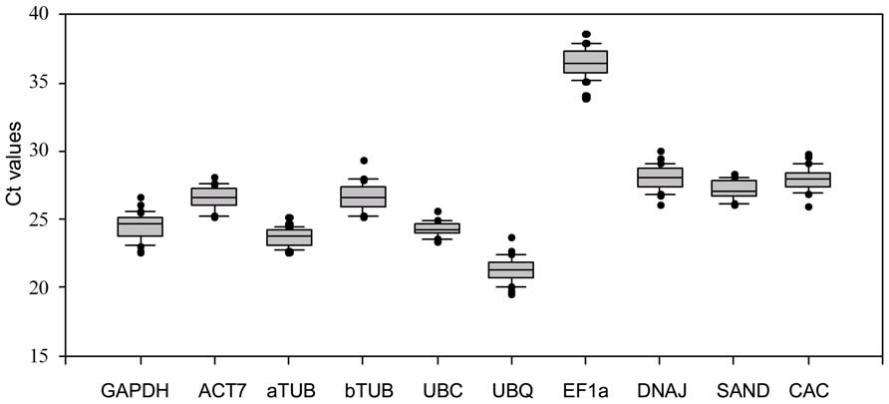
**Expression levels of candidate reference genes in different plant samples.**

In this study, we used qRT-PCR to examine variations in the expressions of 10 candidate reference genes, including 8 traditional housekeeping genes and 2 novel reference genes selected from transcriptome data of *P. orientalis*. Then, we compared their stabilities across a large set of *P. orientalis* samples representing different developmental stages, organs, and stress treatments using statistical and graphical methods. The results demonstrated that the expressions of the selected genes showed different degrees of variations among samples. Furthermore, we examined the expression of a *NAC* transcription factor, a homolog of the stress- and senescence-responsive gene *AtNAC2* [43]–[47], in *P. orientalis* subjected to various stress treatments, using *UBC* and *aTUB* in combination as internal control genes. This work will benefit future studies on gene expression in *P. orientalis* and other members of the Cupressaceae.

## Results

### Expression Profiles of Reference Genes

We selected 10 candidate reference genes, including 8 traditional genes (*GAPDH*, *ACT7*, *aTUB*, *bTUB*, *UBC*, *UBQ*, *EF1a* and *DNAJ*) and 2 novel candidate reference genes (*SAND* and *CAC*) (Table 1, Text S1). The stability of gene expression was determined by quantifying the mRNA level by qRT-PCR. For each gene, we calculated the cycle threshold (Ct) value, which represents the cycle at which a significant increase of the PCR product occurs. In general, this is marked by the middle of the exponential phase of amplification [48], [49]. The expression levels of these 10 reference genes varied widely with Ct values ranging from 19 to 39 cycles (Fig. 1), and most of the Ct values were between 23 and 27 cycles. *UBQ* was the most abundantly transcribed with Ct values of less than 24 cycles; *aTUB*, *UBC* and *GAPDH* were moderately expressed mRNAs with most of the Ct values between 25 and 27 cycles; *ACT7*, *SAND*, *CAC* and *DNAJ* showed Ct values between 26 and 30 or slightly higher. However, *EF1a* showed the lowest level of expression in all samples with Ct values as high as 39 cycles. The calculated coefficient of variance (CV) of the Ct values gives an indication of the expression stability of a particular gene. A narrow range of CV values indicates that a given gene is expressed stably in different samples. Among the 10 candidate reference genes in this study, *EF1a* showed much greater variations in its expression levels than the other genes, whose CV value was more than 5 cycles, whereas *UBC* and *aTUB* showed narrow mean Ct value ranges in their respective expressions with minimal CV. Thus, it is essential to select a set of reliable reference genes to normalize gene expression under certain conditions to obtain accurate gene expression data.

### GeNorm Analysis

**Figure 2:**
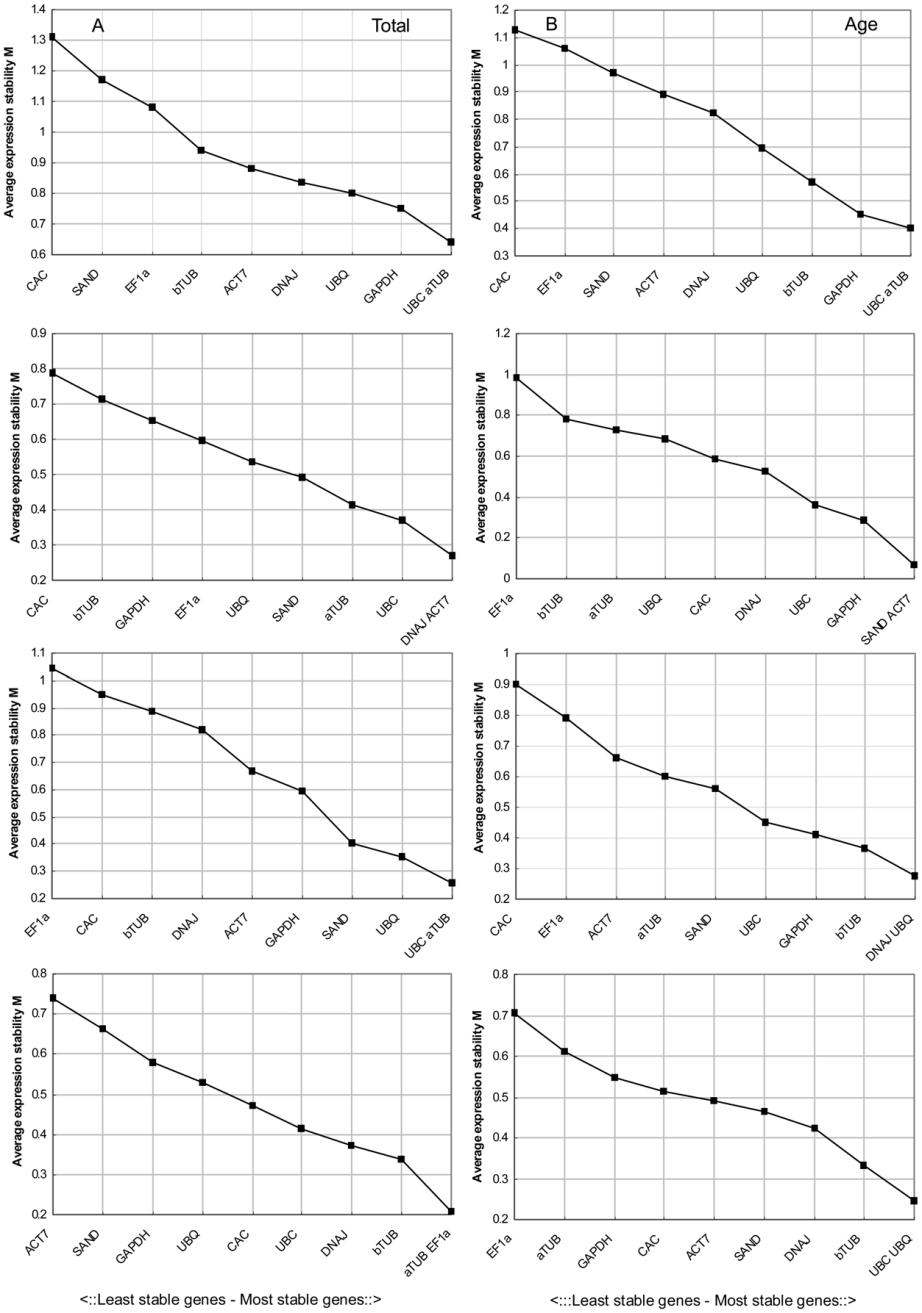
Gene expression stability and ranking of 10 reference genes as calculated by geNorm.

We analyzed gene expression stabilities of the 10 reference genes in all of the designated conditions as described by Vandesompele *et al.* [13] using geNorm software. GeNorm automatically calculates the average expression stability value (M) as the average pairwise variation (V) of a particular gene with all other control genes and determines the V values with all other control genes as the standard deviation of the logarithmically transformed expression ratios [26]. The gene with the lowest M value is that with the most stable expression, while the gene with the highest M value has the least stable expression. As shown in Figure 2, we analyzed data from seven sets of treatments. When all the results from all 29 samples of *P. orientalis* were combined, *aTUB* and *UBC* showed the lowest M value (0.64) and *CAC* showed the highest M value (1.31). Therefore, *aTUB* and *UBC* had the most stable expressions, and *CAC* the least stable expression (Fig. 2A). Among the tissues of different ages, the most stably expressed genes were *UBC* and *aTUB*, while *CAC* was the least stably expressed, consistent with the pattern observed across all samples (Fig. 2B). For the different organs, the *DNAJ* and *ACT7* genes showed the greatest stability of expression, and *CAC* showed the least stable expressions (Fig. 2C). Similarly, the stabilities of reference genes varied among samples under different stress treatments. As shown in Figure 2D, *SAND* and *ACT7* were the most stably expressed under cold stress, and *EF1a* the least stably expressed. In heat-treated samples, *UBC* and *aTUB* were expressed more stably than the other eight reference genes, while *EF1a* was the least stably expressed (Fig. 2E). Under NaCl stress, *DANJ* and *UBQ* were the most stably expressed genes, while *EF1a* and *CAC* were the least stably expressed genes (Fig. 2F). In contrast, under PEG stress, *aTUB* and *EF1a* were the most stably expressed genes and *ACT7* was the most variable (Fig. 2G). The exogenous application of ABA had the least effect on expressions of *UBC* and *UBQ*, and the greatest effect on expression of *EF1a* (Fig. 2H). The geNorm analysis indicated that *EF1a* and *CAC* were the least stably expressed reference gene, although *EF1a* was stably expressed in PEG-treated samples, which is consistent with its roles in stress responses and development. Overall, all of the tested reference genes showed relatively high stability with low M values of less than 1.35, which is below the default limit of M≤1.5. Evaluation of all expression data revealed that *UBC* and *aTUB* were the most stably expressed genes; therefore, these may be suitable reference genes for analyses of gene expression in a wide variety of tissue types, developmental stages, and stress treatments in *P. orientalis* (Fig. 2).

**Figure 3:**
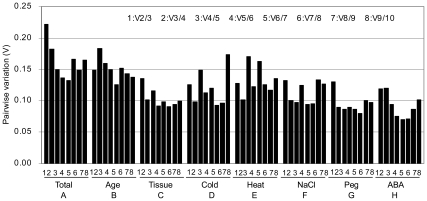
Determination of the optimal number of reference genes for normalization by pairwise variation (V) using geNorm.

To obtain reliable results from RT-PCR studies, it is recommended that two or more reference genes be used. Therefore, Vandesompele *et al*. [13] proposed 0.15 as the cut-off value for V, below which the inclusion of an additional control gene is not required; that is, if Vn/n+1 < 0.15, it is not necessary to use ≥ n+1 reference genes as internal controls. The paired variable coefficients (V2/V3) shown in Fig. 3B, C, D, E, F, G and H indicate that the inclusion of the third reference gene did not contribute significantly to the variation of the normalization factor (V2/3 < 0.15). That is, the two most stable reference genes for each subset would be sufficient for accurate normalization. When all the samples were pooled for analysis, the pairwise variation of V2/3 and V3/4 was greater than 0.15 (0.247 and 0.182, respectively), while that of V4/5 was 0.149 (Fig. 3A), which indicated that four reference genes, *UBC*, *aTUB*, *GAPDH* and *UBQ*, were necessary to normalize gene expression for all the treatments of *P. orientalis* in this study.

**Table 2:** Ranking of candidate reference genes in order of their expression stability as calculated by BestKeeper.

Rank	All(A)	Age(B)	Tissue(C)	Cold(D)	Heat(E)	NaCl(F)	PEG(G)	ABA(H)
1	*UBC*	*aTUB*	*UBC*	*UBQ*	*aTUB*	*GAPDH*	*DNAJ*	*SAND*
CV±SD	1.58±0.38	1.50±0.35	1.50±0.36	0.45±0.10	0.89±0.22	0.43±0.11	0.93±0.26	0.53±0.14
2	*SAND*	*UBC*	*DNAJ*	*aTUB*	*UBC*	*aTUB*	*UBC*	*ACT7*
CV±SD	2.02±0.55	2.10±0.51	1.59±0.43	1.45±0.35	1.02±0.21	0.43±0.29	1.17±0.51	0.79±0.10
3	*CAC*	*SAND*	*ACT7*	*EF1a*	*UBQ*	*UBC*	*SAND*	*UBC*
CV±SD	2.22±0.62	2.15±0.59	1.60±0.41	1.64±0.58	1.21±0.26	0.44±0.11	1.19±0.32	0.82±0.20
4	*aTUB*	*DNAJ*	*aTUB*	*DNAJ*	*SAND*	*DNAJ*	*bTUB*	*aTUB*
CV±SD	2.42±0.57	2.43±0.69	1.73±0.40	1.97±0.56	1.45±0.39	1.01±0.28	1.43±0.37	1.23±0.29
5	*ACT7*	*bTUB*	*SAND*	*UBC*	*GAPDH*	*SAND*	*EF1a*	*DNAJ*
CV±SD	2.56±0.68	2.65±0.70	1.75±0.47	1.97±0.49	1.59±0.40	1.28±0.35	1.54±0.56	1.25±0.35
6	*EF1a*	*ACT7*	*EF1a*	*ACT7*	*ACT7*	*bTUB*	*aTUB*	*bTUB*
CV±SD	2.57±0.94	2.76±0.73	1.87±0.69	2.13±0.57	1.86±0.50	1.81±0.49	1.82±0.42	1.28±0.34
7	*DNAJ*	*GAPDH*	*CAC*	*SAND*	*EF1a*	*UBQ*	*CAC*	*UBQ*
CV±SD	2.65±0.74	2.97±0.73	1.90±0.51	2.2±0.62	2.58±0.95	1.99±0.44	2.20±0.60	1.76±0.38
8	*UBQ*	*EF1a*	*bTUB*	*GAPDH*	*CAC*	*EF1a*	*ACT7*	*CAC*
CV±SD	2.97±0.63	3.22±1.16	2.22±0.58	2.52±0.64	3.47±0.96	2.21±0.80	2.27±0.61	1.86±0.51
9	*bTUB*	*CAC*	*UBQ*	*bTUB*	*DNAJ*	*ACT7*	*UBQ*	*GAPDH*
CV±SD	3.09±0.82	3.40±0.95	2.65±0.55	2.85±0.79	3.55±1.00	2.48±0.66	3.3±0.69	1.99±0.49
10	*GAPDH*	*UBQ*	*GAPDH*	*CAC*	*bTUB*	*CAC*	*GAPDH*	*EF1a*
CV±SD	3.18±0.78	3.97±0.85	2.92±0.70	3.21±0.89	3.78±1.02	3.73±1.04	3.48±0.83	2.74±1.01

Note: Expression stability and ranking of 10 reference genes as calculated by Bestkeeper in all samples (A), different ages (B), different tissue types (C), cold-treated (D), heat-treated (E), NaCl-treated (F), PEG-treated (G), ABA-treated (H). Descriptive statistics of 10 candidate genes based on their coefficient of variance (CV) and standard deviation (SD) of Ct values were determined using the whole data set, and all Ct values were analyzed as a total data set. Reference genes are identified as the most stable genes (those with the lowest coefficient of variance and standard deviation; CV±SD).

### BestKeeper Analyses

For analyses using BestKeeper, an Excel-based tool, the average Ct value of each duplicate reaction is used (without conversion to quantity) to analyze the stabilities of candidate reference genes [50]. BestKeeper evaluates the stabilities of candidate reference genes based on the coefficient of correlation to the BestKeeper index, which is the geometric mean of the Ct values of all candidate reference genes [50], [51]. BestKeeper also calculates the coefficient of variance (CV) and the standard deviation (SD) of the Ct values using the whole data set and all the Ct values are analyzed as a total data set [51]. Reference genes are identified as the most stable genes, as they exhibit the lowest coefficient of variance and standard deviation (CV±SD). Genes that show a SD greater than 1 are considered unacceptable [52]. In this study, *UBC* and *SAND* had CV±SD values of 1.58±0.38 and 2.02±0.55, respectively, and showed remarkably stable expression in all the samples. However, *GAPDH* and *bTUB* had CV±SD values of 3.18±0.78 and 3.09±0.82, respectively, and showed the least stable expression (Table 2). These results differed to those obtained using geNorm (Fig. 2). For the different ages of *P. orientalis*, the most stable reference genes (lowest CV±SD) were *aTUB* and *UBC*, while *UBQ* had the highest CV±SD of all the selected genes. Bestkeeper analyses indicated that *UBC* and *DNAJ* were the most stably expressed, and *GAPDH* and *UBQ* were the least stably expressed among different tissue types and PEG-treated samples. *UBQ* and *aTUB* were the most stably expressed genes under cold stress; and *UBC* and *aTUB* had the lowest coefficient of variance under heat treatment, which was consistent with the results obtained using geNorm and NormFinder. In ABA-treated samples, BestKeeper analysis indicated that the most stable genes were *SAND* and *ACT7* and the least stable were *GAPDH* and *EF1a.* The results obtained from BestKeeper showed slight differences from those obtained from geNorm (Fig. 2).

### NormFinder Analysis

Similar to geNorm, the NormFinder program is a Visual Basic application tool for Microsoft Excel used to determine expression stabilities of reference genes [53]. As in the geNorm method, the gene with the lowest M value is that with the most stable expression, and the gene with the highest M value has the least stable expression. The results of the NormFinder analysis were showed in Table 3. The NormFinder outputs with and without different sample subgroups showed some common features. The NormFinder analysis ranked *UBC* and *aTUB* in the top positions for all the samples, different age samples, and heat-treated samples, while *EF1a* and *CAC* were ranked as less stable, consistent with the result obtained from geNorm. Among the different tissues, *DNAJ* and *ACT7* were the most stably expressed with values of 0.093 and 0.101, respectively, while expressions of *CAC* and *bTUB* were the least stable. Under cold, ABA, and NaCl treatments, *UBC* was calculated to be the most stably expressed gene, and *EF1a* was the least stable. Under PEG treatment, *bTUB* and *aTUB* were predicted as the best internal controls, while *ACT7* and *SAND* were the least stably expressed genes. The results obtained from NormFinder were highly consistent with those obtained from geNorm (Fig. 2).

**Table 3:** Ranking of candidate reference genes in order of their expression stability as calculated by NormFinder.

Rank	All(A)	Age(B)	Tissues(C)	Cold(D)	Heat(E)	NaCl(F)	PEG(G)	ABA(H)
1	*UBC*	*aTUB*	*DNAJ*	*UBC*	*aTUB*	*UBC*	*bTUB*	*UBC*
M value	0.263	0.099	0.093	0.139	0.058	0.067	0.094	0.082
2	*aTUB*	*UBC*	*ACT7*	*ACT7*	*UBC*	*GAPDH*	*aTUB*	*UBQ*
M value	0.362	0.270	0.101	0.227	0.089	0.067	0.121	0.232
3	*UBQ*	*GAPDH*	*aTUB*	*SAND*	*UBQ*	*SAND*	*DNAJ*	*SAND*
M value	0.533	0.351	0.273	0.292	0.180	0.251	0.155	0.245
4	*GAPDH*	*bTUB*	*UBC*	*DNAJ*	*SAND*	*aTUB*	*UBC*	*CAC*
M value	0.553	0.494	0.279	0.388	0.226	0.259	0.228	0.262
5	*ACT7*	*ACT7*	*SAND*	*GAPDH*	*ACT7*	*DNAJ*	*EF1a*	*DNAJ*
M value	0.591	0.594	0.312	0.402	0.495	0.352	0.237	0.264
6	*bTUB*	*UBQ*	*EF1a*	*UBQ*	*GAPDH*	*UBQ*	*CAC*	*ACT7*
M value	0.603	0.663	0.395	0.415	0.509	0.409	0.361	0.274
7	*DNAJ*	*DNAJ*	*UBQ*	*CAC*	*CAC*	*bTUB*	*UBQ*	*bTUB*
M value	0.608	0.678	0.450	0.467	0.696	0.522	0.479	0.366
8	*SAND*	*SAND*	*GAPDH*	*aTUB*	*DNAJ*	*ACT7*	*GAPDH*	*GAPDH*
M value	0.715	0.680	0.552	0.487	0.781	0.635	0.523	0.391
9	*EF1a*	*EF1a*	*bTUB*	*bTUB*	*bTUB*	*EF1a*	*SAND*	*aTUB*
M value	0.852	0.810	0.572	0.665	0.795	0.757	0.547	0.519
10	*CAC*	*CAC*	*CAC*	*EF1a*	*EF1a*	*CAC*	*ACT7*	*EF1a*
M value	1.126	0.937	0.673	1.199	0.923	0.871	0.662	0.689

Note: Expression stability and ranking of 10 reference genes as calculated by NormFinder in all samples (A), different ages (B), different tissue types (C), cold-treated (D), heat-treated (E), NaCl-treated (F), PEG-treated (G), ABA-treated (H). Lower average expression stability (M value) indicates more stable expression.

### Evaluation of Reference Genes for Determining Age- and Stress-responsive *NAC* Expression

**Figure 4:**
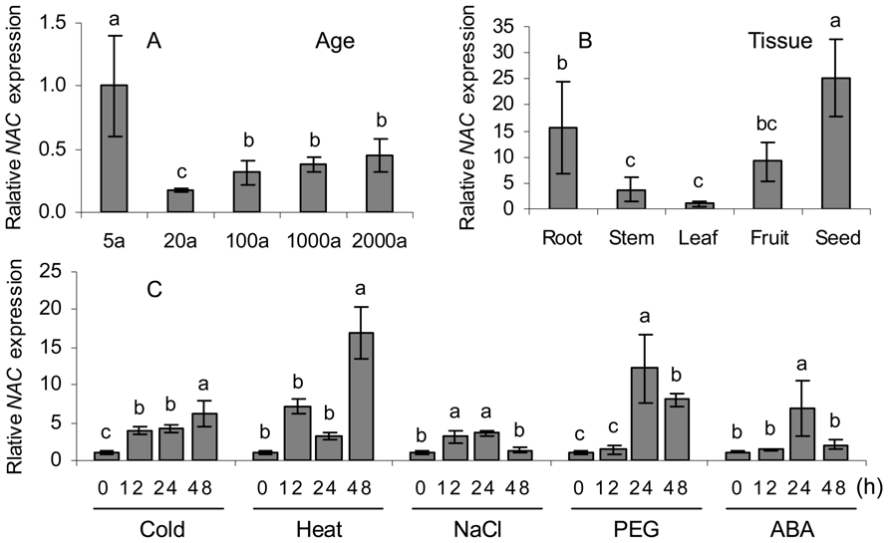
Expression profiles of *NAC* in different-aged tissues and in response to stresses in *Platycladus orientalis* (as determined by qRT-PCR with UBC and aTUB in combination as reference genes).

To further evaluate the reliability of the top two reference genes *aTUB* and *UBC*, *NAC* transcription factor *AtNAC2* (At5g39610; also named *ANAC092*), which plays a crucial role in protecting plants against abiotic stresses, as well as in leaf senescence [45], was selected to qRT-PCR analysis with *aTUB* and *UBC* singly or in combination as reference genes. In our previous study, we found that the NAC domain gene from *P. orientalis*, a homolog of *AtNAC2* in *A. thaliana*, was differentially expressed between young and old trees by using RNA-seq combined with a DGE technique (data not shown). Therefore, this gene was selected to further evaluate the reliability of the reference genes by qRT-PCR. We selected *aTUB* and *UBC* in combination as the reference genes and determined their average expression patterns (Fig. 4). The highest level of *NAC* expression was in leaves of 5-year-old *P. orientalis*. The expression of *NAC* in leaves increased significantly from 20- to 2000-year-old individuals of *P. orientalis* (*P* < 0.05) (Fig. 4A), which matches the result obtained from the DGE analysis. *NAC* showed evidently different expression levels in the tissues, such as seeds, fruits, roots, stems, and leaves (*P* < 0.05) (Fig. 4B). Cold and heat stress significantly increased the expression of *NAC* (Fig. 4C) by 8.0 and 5.7 folds, respectively, at 48 h compared with that at 0 h (*P* < 0.05). The expression profiles of *NAC* showed similar trends under NaCl, PEG, and ABA treatments, and its abundance increased to maximum levels at 24 h, increasing to 12.9, 5.6 and 12.2 folds compared with that at 0 h, respectively (*P* < 0.05) (Fig. 4C). In addition, the expression patterns of *NAC* showed similar trends to those of *aTUB* and *UBC* as internal controls either singly or in combination (Fig. 4; Fig. S1). There were no significant differences in the expression patterns of *NAC* using either *aTUB* or *UBC* singly as the internal control (*P* > 0.05), although it seems that there was a bit of difference between *aTUB* and *UBC* at the 48-hour treatments (*P*  =  0.059) (Fig. S1), which further indicated that *aTUB* and *UBC* were suitable as reference genes.

## Discussion

In plant molecular biological research, studies on gene expression patterns help us to understand biological processes. The qRT-PCR technique is one of the most common methods to quantify gene expression levels, which is a crucial step in identifying gene function [26]. However, to accurately analyze expression of a particular gene, it is essential to have a reliable method to normalize its expression. Thus, an appropriate internal reference gene is required for reliable quantification of gene transcripts.

To screen for appropriate reference genes suitable for studies on age- and stress-responsive gene expression in *P. orientalis*, we examined the expressions of 10 reference genes from its transcriptome in a set of different conditions. We then analyzed their expressions using three different software packages: geNorm, NormFinder and Bestkeeper. Analysis with geNorm is an easy method to determine the optimal number of stable housekeeping genes for accurate normalization [29], whereas NormFinder and Bestkeeper were used to assess the quality of the ranking obtained by geNorm [54]. Our data showed that the top two positions of reference genes for all samples, for samples of different ages and different tissue types, and for heat-treated samples, were almost the same when determined by geNorm, Bestkeeper, and NormFinder. The top two positions of reference genes in the cold-, PEG- and ABA-treated samples determined by geNorm were highly similar to those determined by NormFinder but not Bestkeeper. The top two positions of reference genes in the NaCl-treated samples predicted by NormFinder were similar to those determined by Bestkeeper but not geNorm (Table S1). The three software packages use different calculation algorithms [55] and therefore can give different results. However, all three software packages showed that *aTUB* and *UBC* were the most stable reference genes. In addition, different types of samples had their own best reference genes among the 10 selected candidate reference genes (Fig. 2; Table 2, 3). For example, *DNAJ* was one of the best reference genes for different tissues types and NaCl-treated samples, whereas *UBQ* performed better than *ACT7* as a reference gene for NaCl- and ABA-treated samples. In general, *UBC* and *aTUB* were the optimum pair of reference genes for all the samples, all developmental stages, and in heat-treated samples. Therefore, it is necessary to validate the expression stability of the control gene under specific experimental conditions prior to its use for normalization [26].

Our data demonstrate that *UBC*, *aTUB*, *DNAJ* and *ACT7* were ranked in top positions in all the samples of *P. orientalis* based on the results from the three software packages. Here, *UBC* was found to be one of the most stably expressed genes in all samples of *P. orientalis*, which was consistent with the result in *B. distachyon* [26], whereas it showed less stable expression in *A. thaliana* under heavy metal (Cu and Cd) stress [23], therefore expression levels of reference genes vary among different species [20]. In this study, *aTUB* expression did not vary, or varied little, among tissues of different ages and under PEG stress (Fig. 2; Table 2), *aTUB* was also identified as being stably expressed across various developmental stages of soybean [29] and different tissues of poplar [34]. *DNAJ* showed remarkably stable expression in the different tissues and NaCl-treated samples of *P. orientalis*, which was consistent with an earlier study on *DNAJ* expression in tomato [56]. As noted previously, expression of *ACT7* is relatively weak in soybean, rice, potato, and sugarcane [23]–[25], and rather variable in *A. thaliana* [16]. In our study, geNorm analysis indicated that expression of *ACT7* was most stable across different tissues and cold-treated samples of *P. orientalis*.

All three software packages indicated that *UBQ*, *bTUB*, and *GAPDH* ranked in middle positions in all samples of *P. orientalis*. In the present study, *UBQ* was expressed stably in NaCl- and ABA-treated samples. *UBQ* also showed very stable expression in *A. thaliana* and *B. distachyon* [26], but was unsatisfactory as a reference gene in soybean [29] and grape [57]. *GAPDH* is one of the most commonly used reference genes to normalize gene expression data in qRT-PCR assays [58]. Here, we found that *GAPDH* was the most stably expressed reference gene in different ages, but was expressed less stably in different tissues of *P. orientalis*. *bTUB* was reported to perform poorly as a reference gene in grape and potato [32], [58], while in this study, NormFinder analysis indicated that *bTUB* showed highly stable expression in PEG-treated samples of *P. orientalis*.

Previously, *EF1a* was reported to be stably expressed during biotic and abiotic stress in both potato and rice [29], [32]. In this study, however, analyses using all three software packages ranked *EF1a* in the bottom positions. Similarly, the novel reference gene *CAC* was the lowest ranked gene in analyses from geNorm and NormFinder in all samples. These results indicated that *EF1a* and *CAC* were both unsuitable reference genes in all *P. orientalis* samples. Additionally, the novel reference gene *SAND* was not the best choice to analyze *P. orientalis* gene expression over a wider range of conditions, although it was stably expressed under cold stress in *P. orientalis*. However, *SAND* and *CAC* were the recommended reference genes for studies on development in berry [58] and tomato [31], respectively. Therefore, to investigate the transcript stability of the commonly used reference genes and to identify novel and superior reference genes, it is necessary to collect as many data as possible about gene expression in different organisms, organs, and experimental conditions.

To further validate the applicability of the screened reference genes, we analyzed the expression of the NAC domain gene, a homolog *AtNAC2* of *A. thaliana*, in *P. orientalis. AtNAC2* has recently been discovered as a central regulator of age-dependent and salt-promoted senescence in *A. thaliana* [45]. In this study, we examined the expression of *NAC* using a combination of *UBC* and *aTUB* as reference genes. The result showed that *NAC* exhibited a leaf age-dependent expression pattern with low to moderate expression in 20-year-old tree leaves, and significantly high expression in leaves of 1000- and 2000-year-old individuals of *P. orientalis* (*P* < 0.05). This pattern of expression was highly similar to that obtained from the DGE profile (data not shown), and the changes in the expression patterns of *NAC* under all the designated treatments (Fig. 4) showed similar trends to those of *aTUB* and *UBC* under the same conditions (Fig. S1). However, because subtle changes in expression are of critical importance in some experiments, it may not be suitable to use a single reference gene [13]. Therefore, to understand the molecular mechanisms underlying the stress tolerance and longevity of *P. orientalis*, it would be helpful to use two or more reliable reference genes for normalization of expression of genes of interest.

Above all, we identified 10 reference genes that were suitable for normalization of qRT-PCR data obtained from *P. orientalis* samples of different ages, from different tissues, and from plants subjected to various exogenous treatments. Evaluations using geNorm, NormFinder and BestKeeper indicated that the two most suitable reference genes in *P. orientalis* were *aTUB* and *UBC*, while the two least suitable reference genes were *EF1a* and *CAC*. To obtain the most reliable results from gene expression studies of *P. orientalis*, it is recommended that two or more reference genes are used as internal controls for relative gene quantification.

## Materials and Methods

### Plant Materials and Treatments

The trees sampled in this study were 20-, 100-, 1000-, and 2000-year-old individuals of *P. orientalis* growing in similar conditions in Zhongshan Park, Beijing. Nine-month-old and 5-year-old seedlings of *P. orientalis* were cultivated in pots with soil in the greenhouse at the Chinese Academy of Forestry, Beijing. For salt-, osmotic-, and ABA-treatments, 9-month-old seedlings were carefully pulled out from pots, washed cleanly with tap water, and placed in the solutions of NaCl (200 mM), PEG6000 (10%), and ABA (150 µM), respectively, for 0, 12, 24, and 48 h in the greenhouse. For the cold- and heat-treatments, the seedlings in pots were placed at 4°C or 40°C, respectively, in chambers with a 14-h light/10-h dark photoperiod for 0, 12, 24, and 48 h.

For the collection of tissues of trees in 20-, 100-, 1000-, and 2000-year-old, fresh leaves were collected from the branches in different directions in June and August 2011. Different tissues including leaves, roots, stems, fruits, and seeds were collected from 5-year-old seedlings. Leaves from 9-month-old seedlings were collected from the whole seedlings subjected to various treatments, immediately frozen in liquid nitrogen and stored at -80°C. Samples above were collected from 3 trees to give 3 replicas.

### Total RNA Extraction and cDNA Synthesis

Total RNA was extracted from treated samples using a Column Plant RNAout kit (TIANDZ, CHINA), and then genomic DNA and polysaccharides were eliminated using RNase-free DNase I (TIANDZ, CHINA) and Polysaccharide Erasol (TIANDZ, CHINA) kits, respectively. The purity of the total RNA extracted was determined using a NanoDrop DU8000 spectrophotometer. RNA samples with an absorbance ratio at OD260/280 between 1.9 and 2.2 and OD260/230 ≈ 2.0 were used for further analyses. RNA integrity was verified by 2% agarose gel electrophoresis and ethidium bromide staining. Samples with 28S/18S ribosomal RNA between 1.5 and 2.0 and without smears on the agarose gel were used for the following experiment. First-strand cDNA was synthesized from 600 ng total RNA in a volume of 20 µl using the PrimeScript® RT reagent kit (TaKaRa, Japan) according to the manufacturer’s protocol. cDNA was diluted 7.5 folds before quantification and determinations of quantity and quality.

### Quantitative Real-time RT-PCR

The primers for the 10 reference genes from the transcriptome of *P. orientalis* were designed using Primer 3 software (http://frodo.wi.mit.edu/primer3/). All primer pairs were initially tested via standard RT-PCR using the Premix Ex Taq (TaKaRa, Japan) and a single amplification product of the expected size for each gene was verified by electrophoresis on a 3% agarose gel and staining with ethidium bromide. qRT-PCR reactions were carried out in 96-well blocks with an Applied Biosystems 7500 Real-Time PCR system using SYBR® Premix Ex Taq^TM^ (TaKaRa, Japan) in a 20 µl reaction volume (containing 2 µl cDNA reaction mixture, 10 µl 2×SYBR Premix Ex TaqTM, 0.4 µl ROX Reference Dye II, and 0.4 µl each primer). The reaction conditions were those recommended by the manufacturer (30 s at 95°C, 40 cycles of 95°C for 5 s, and 60°C for 34 s). The dissociation curve was obtained by heating the amplicon from 60 to 95°C. All qRT-PCR reactions were carried out in technical and biological triplicate. The final threshold cycle (Ct) values were the mean of nine values (biological triplicate, each in technical triplicate).

### Statistical Analyses

To select a suitable reference gene, the stability of mRNA expression of each reference gene was statistically analyzed with three different types of Microsoft Excel-based software: geNorm [59], NormFinder [60], and BestKeeper [61]. All three software packages were used according to the manufacturer’s instructions. For geNorm and NormFinder, the raw Ct values were transformed into the required data input format. The maximum expression level (the lowest Ct value) of each gene was used as a control and was set to a value of 1. Relative expression levels were then calculated from Ct values using the formula: 2^-△Ct^, in which △Ct = each corresponding Ct value–minimum Ct value. The obtained data were further analyzed with geNorm and NormFinder. BestKeeper analyses were based on untransformed Ct values.

Standard curves were generated using Excel software by plotting cycles at threshold fluorescence (Ct) against the logarithmic values of standard RNA amounts. Quantities of standard RNA were prepared by diluting 200 ng cDNA (1, 1/5, 1/25, 1/125, 1/625, 1/3125; each gene in triplicate). Only Ct values of less than 40 were used to calculate correlation coefficients (r^2^ values) and amplification efficiencies (E) from the given slope generated in Microsoft Excel 2003 according to the equation E =  [5^-(1/ slope)^ – 1]×100%. All PCR assays showed efficiency values between 95 and 105%.

Data were compared and analysed with analysis of variance (ANOVA) and multiple comparisons using the statistical analysis software of SPSS. Differences were scored as statistical significance at the *P* < 0.05 or *P* < 0.01 level.

## Supporting Information

Text S1 **A list of sequences of candidate housekeeping genes and NAC domain protein gene.**
(DOC)

Figure S1 **The expression profile of** ***NAC*** **responsive to aging and stresses in** ***Platycladus orientalis*** **(studied by qRT-PCR with** ***UBC*** **and** ***aTUB*** **as reference genes, respectively).**
(DOC)

Table S1 **The ranking of 10 reference genes and the assembly of the comparisons in different samples of** ***Platycladus orientalis*** **as calculated by geNorm, Bestkeeper, and NormFinder**.
(DOC)
