# Supplementary material for: Selection of Reference Genes for Quantitative Gene Expression Studies in Platycladus orientalis (Cupressaceae) Using Real-Time PCR
Source: PLoS One. 2012 Mar 30;7(3):e33278. doi: 10.1371/journal.pone.0033278 (PMC3316566; doi:10.1371/journal.pone.0033278)
Supplement: Text S1 — A list of sequences of candidate housekeeping genes and NAC domain protein gene. (DOC) [file pone.0033278.s001.doc]

**Text S1. A list of sequences of candidate housekeeping genes and *NAC* domain protein gene.**

***GAPDH* glyceraldehyde-3-phosphate dehydrogenase (150bp) (*Arabidopsis* homolog locus AT1G79530)**

1 GAGATTCCAT GGGGTGATTT TGGAGCTGAC TATGTTGTAG AGTCATCTGG TGTATTTACC

61 ACAACTGAAA AGGCATCTGC ACATCTTAAG GGTGGTGCCA AAAAGGTTGT AATTTCTGCA

121 CCGTCAGCAG ATGCCCCTAT GTTTGTGGTT 150

***ACT7* actin 7 (516 bp) (*Arabidopsis* homolog locus AT5G09810)**

1 GCAGAACGAG AAATTGTCAG AGACATAAAG GAAAAATTGG CTTATGTTGC AATGGATTAT

61 GAAAAGGAGT TGGAACTATC AAGAAGCGAA GCAACATTAG AAAAGAGTTA CGAGCTTCCA

121 GATGGTCAAG CAATCACAAT TGGAAATGAA AGGTTCAGAT GTGCCGAGGT GTTGTTCCAG

181 CCTTCTCTCA TTGGTATGGA GGCTCCGGGA ATACATGAAA CTGCTTTTGC TTCCATCATG

241 AAATGTGATG TGGACATTAG AAAGGACTTG TATGGAAACA CTGTTCTCAG TGGAGGTTCC

301 ACCATGTTTC CTGGTATTGC TGACCGCATG AGTAAAGAAA TTGCAGCTCT TGCTCCGAAT

361 AGCATGAAAG TTAAGGTGGT TGCCCCTCCT GAGAGAAAAT ACAGTGTCTG GATTGGAGGC

421 TCCATTCTCG CTTCGCTCAG CACATTCCAA CAGATGTGGG TAACAAAGGC TGAATATGAT

481 GAGTCTGGAC CTTCAATTGT TCACAGGAAA TGCTTC 516

***aTUB* alpha-tubulin (240 bp) (*Arabidopsis* homolog locus** [**AT5G19770**](http://arabidopsis.org/servlets/TairObject?type=locus&name=AT5G19770)**)**

1 TCTTCACTGA CCACATCTCT TAGGTTTGAT GGAGCAATTA ATGTGGATGT GACAGAATTT

61 CAGACAAATC TTGTACCATA TCCTAGAATC CACTTCATGC TCTCCTCCTA TGCTCCAGTA

121 ATTTCTGCAG AGAAAGCTTA CCATGAGCAA TTATCTGTTC CTGAGATCAC AAATGCAGTA

181 TTTGAACCTT CCAGCATGAT GGCCAAGTGT GACCCCAGAC ATGGAAAATA TATGGCTTGC 240

***bTUB* beta tubulin (714 bp) (*Arabidopsis* homolog locus AT1G20010)**

1 ATGAGAGAAA TTCTGCATAT TCAGGGCGGG CAGTGTGGTA ATCAAATTGG GGCAAAGTTC

61 TGGGAAGTCA TTTGCGATGA ACACGGGATC GATCCGACGG GGTCGTACAA GGGCGACTCC

121 GATCTGCAGC TGGAGAGAAT CAATGTGTAT TATAATGAGG CCAGTGGAGG CCGGTACGTG

181 CCCCGAGCGG TGCTAATGGA TCTCGAACCC GGGACCATGG ACAGTGTTAG ATCTGGTCCT

241 TATGGTCAGA TTTTTAGGCC TGATAACTTC GTCTTCGGCC AGACTGGTGC TGGAAATAAC

301 TGGGCCAAGG GACATTACAC CGAGGGTGCG GAGCTTATCG ACTCTGTTCT GGATGTTGTT

361 CGCAAGGAGG CCGAGAATTG CGATTGTCTT CAAGGATTTC AAGTTTGCCA TTCTTTGGGG

421 GGAGGCACAG GATCAGGAAT GGGAACTCTC TTGATCTCCA AAATCAGGGA GGAGTATCCA

481 GATAGAATGA TGTTAACTTT CTCTGTTTTC CCATCGCCTA AGGTATCGGA CACAGTGGTT

541 GAGCCTTACA ATGCAACTCT GTCTGTTCAT CAATTAGTAG AGAATGCAGA CGAATGCATG

601 GTCCTGGACA ATGAAGCGCT TTATGATATT TGCTTCAGAA CATTAAAGCT AACAACGCCT

661 ACATTTGGAG ATCTTAACCA TTTAATATCT GCGACAATGA GCGGATGCAC TTGTTGCCTT

721 AGGTTCCCTG GACAATTAAA C 714

***UBC* ubiquitin-conjugating enzyme E2 (246 bp) (*Arabidopsis* homolog locus AT3G57870)**

1 ATGTCCGGCG GTATTGCTCG TGGTCGTCTT GCTGAAGAGC GGAAGGCGTG GCGGAAGAAT

61 CACCCGCACG GTTTTGTTGC CAGGCCTGAT TCTCAACCAG ATGGCTCCTT GAACTTGATG

121 GTCTGGCAGT GCATCATTCC AGGAAAAGCT GGGACTGACT GGGAGGGTGG ATACTTTCCA

181 CTAGCAATCC ATTTCAGTGA GGATTATCCA AGTAAACCAC CAAAGTGCAA GTTTCCACAA

241 GGTTTT 246

***UBQ* ubiquitin 10.2 (261 bp) (*Arabidopsis* homolog locus** [**AT5G20620**](http://arabidopsis.org/servlets/TairObject?type=locus&name=AT5G20620)**)**

1 CCTCCAGACC AGCAAAGGCT GATCTTCGCT GGAAAACAAC TCGAGGATGG ACGCACCCTT

61 GCGGATTACA ATATCCAAAA GGAGTCAACC CTTCACCTGG TTCTCCGTCT GAGGGGAGGC

121 ATGCAGATTT TTGTCAAAAC CCTCACTGGA AAAACCATCA TTCTTGAGGT GGAGAGCTCC

181 GATACCATTG ATAATGTTAA GGCTAAGATA CAGGACAAGG AGGGCATTCC TCCTGACCAG

241 CAGAGGCTGA TCTTCGCTGG C 261

***EF1a* elongation factor 1-alpha (255 bp) (*Arabidopsis* homolog locus AT1G07940)**

1 TACAAGGGTT GGGAGAAGGA GACCAAGGCC AAGGCCACTG GCAAGACCCT CCTCGAGGCC

61 ATTGACGCCA TCGACCCTCC TTCCCGTCCT ACCGACAAGC CTCTCCGTCT GCCCCTTCAG

121 GATGTTTACA AGATCTCCGG TATTGGCACA GTTCCCGTCG GTCGTGTCGA GACCGGTATC

181 ATCAAGGCCG GTATGGTCGT CACCCTCGCC CCCGCTGGTG TCACCACTGA GGTCAAGTCC

241 GTCGAGATGC ACCAC 255

***DNAJ* DnaJ-like protein (369 bp) (*Arabidopsis* homolog locus** [**AT3G44110**](http://arabidopsis.org/servlets/TairObject?type=locus&name=AT3G44110)**)**

1 GACCCAGAAG GTGATCGCCC AGGTGATCTT TATGTGTTAC TCAAGGTCCA TGATGATCCT

61 ATATTTCGCA GAGAAGGAGC TCACATTCAC GTAGATGCAG TTATAAGCTT CACTCAGGCT

121 ATTCTTGGTG GAACCATTCA GGTGCCGACT TTAACTGGTG ATGTGGTTTT GAAGGTTCGT

181 GAAGGCACAC AGCATGGTCA ACAGGTTGTT TTGAAAGGAA AAGGTATTAA GCTACGGAAT

241 TCTAGCCAGT ACGGCAATCA ATATGTACAT TTTCAAGTGA TCATTCCAAC GAACCTGACA

301 CACAGGCAAC GAATGCTTAT AGAGGAATTT GCAAAAGAAG AAAACAGTGA AGATGATAAG

361 AGTGCTGCT 369

***SAND* sand family protein (552 bp) (*Arabidopsis* homolog locus AT2G28390)**

1 TGCCGGATAC GCATTGAGAA TGTACTGGTC AGATCACAAG TCCTAAGTGA GGTTCAAAAA

61 TCTGTGCTAG ATGGTGGTCT GCATGTGGAA GACTTACCTG GTGAGAACAT GGTGCCTATG

121 GGATCAACGT CCTCCCGTTT CGAACAGGAC ATGGTTGGAT CTTCTACAGG AATCGAGAGA

181 AGAAATATTG GAATTGGAGG TCCGGCTGGG TTATGGCATT TTATGTACCG TAGTAACTAT

241 CTGAATCAGT ATGTAGCTTC AGAATATCCA CCACCCTTAA ATAACCGTAA TGCACAGAAG

301 AGGTTATTCA GGGCATATCA AAAATTGTAT GCCTCAATGC ATGATAGGGA TATAGGACCT

361 CACAAGATGC AGTACAGGAA AGATGAAAAC TATGTTCTTT TATGCTGGAT TACTCAGGAT

421 TTTGAGCTTT ATGCAGCTTT TGATCCACTG GCTGAAAAGA GCTCAGCAAT AACTGTTTGC

481 AATAGAGTTT GCCAATGGTT GCGAGACATT GAAAGCGAGA TATTTTTACT AGGAGCAAGC

541 TCACTTTCCT GG 552

***CAC* clathrin adaptor complexes medium subunit family protein (135 bp) (*Arabidopsis* homolog locus** [**AT1G56590**](http://www.arabidopsis.org/servlets/TairObject?type=locus&name=AT1G56590)**)**

1 ATGATGCAAT GTATATTTCT CCTCTCAGAT ACTGGGGAAG TAATGCTTGA GAAGCAATGG

61 ATGGGGCAAC AGGTGGACAG ATCTATATGT GTTTGGTTTT GGGAACAAGC TTTTCCCGTG

121 AGAGATTCTT TCAAG 135

***NAC* NAC domain protein gene (669 bp) (*Arabidopsis* homolog locusc AT5G39610)**

1 CACCCGGATC TACCTCCGGG ATTTCGGTTT CATCCTACTG ATGAAGAGCT GCTGATTCAT

61 TATCTCAGGA AGTCTTCTCC TCTGCCGGTC TCCATTATTG CAGATGTTGA TCTCTATAAG

121 TTTGATCCCT GGGATCTGCC AGGAATGGCA AGATTTGGTG AACACGAGTG GTATTTCTTC

181 AGCCCTAGGG AGCGGAAGTA TCCTAATGGC GCGCGGCCGA ACAGGGCGGC GGCGTCAGGG

241 GACTGGAAGG CCACAGGCAC TGACAAACCC GTGGTCATAT CAGGAACCTC GCACAAGGTA

301 GGCGTGAAGA AAGCTCTAGT TTTCTACAAG GGGAAACCCC CGAAGGGCAT TAAAACGAAC

361 TGGATCATGC ACGAGTATCG CCTCACAGAT TCCACGGACA TTTGCTCGCC TTCGCCCAGG

421 CGAAAAGGAT CTCTGCGACT CGATGACTGG GTGCTTTGTC GAATCTACAA AAAAGTGAGC

481 CATTTTCCGA GAGGAGAAGG AAGCGAAGGG GAGGATTCAA TGGCCGATTC TTTGCCTTCT

541 GCAGGGCAAC ACTCGCATGA AAACATGGAA CCTGCACCGA GATTCGATTC CTTTTCTGAT

601 CTTCTCCAGA TGGAAGATCC AAACTTTCTG GGGGGATATT TGGACTCATC ATACGCCAAC

661 GGGAATCTG 669
