# Supplementary material for: Selection of Reference Genes for Quantitative Gene Expression Studies in Platycladus orientalis (Cupressaceae) Using Real-Time PCR
Source: PLoS One. 2012 Mar 30;7(3):e33278. doi: 10.1371/journal.pone.0033278 (PMC3316566; doi:10.1371/journal.pone.0033278)
Supplement: Figure S1 — The expression profile of NAC responsive to aging and stresses in Platycladus orientalis (studied by qRT-PCR with UBC and aTUB as reference genes, respectively). (DOC) [file pone.0033278.s002.doc]

**Figure S1. The expression profile of *NAC* responsive to aging and stresses in *Platycladus orientalis*** (studied by qRT-PCR with *UBC* and *aTUB a*s reference genes, respectively). Error bars represent the mean ± standard deviation for *n* = 9 (biological triplicate, each with technical triplicate). Statistical analysis was analysed with analysis of variance and multiple comparisons by SPSS software. Lowercase letters indicate statistically signiﬁcant differences among the treatments within different ages, different tissues, cold-, heat-, NaCl-, PEG- and ABA-treated (*P* < 0.05). In tissue samples group R: root, S: stem, L: leaf, F: fruit, Se: seed.

0 12 24 48

0 12 24 48 (h)

*UBC aTUB*

Cold

0 12 24 48

0 12 24 48 (h)

*UBC aTUB*

Heat

0 12 24 48

0 12 24 48 (h)

*UBC aTUB*

ABA

0 12 24 48

0 12 24 48 (h)

*UBC aTUB*

Nacl

0 12 24 48

0 12 24 48 (h)

*UBC aTUB*

Tissues

R S L F Se

*UBC aTUB*

R S L F Se

*UBC aTUB*

20 100 1000 2000(a)

20 100 1000 2000

Age

PEG
