# Supplementary material for: Selection of Reference Genes for Quantitative Gene Expression Studies in Platycladus orientalis (Cupressaceae) Using Real-Time PCR
Source: PLoS One. 2012 Mar 30;7(3):e33278. doi: 10.1371/journal.pone.0033278 (PMC3316566; doi:10.1371/journal.pone.0033278)
Supplement: Table S1 — The ranking of 10 reference genes and the assembly of the comparisons in different samples of Platycladus orientalis as calculated by geNorm, Bestkeeper, and NormFinder. (DOC) [file pone.0033278.s003.doc]

**Table S1. The ranking of 10 reference genes and the assembly of the comparisons in different** **samples of *Platycladus orientalis*** **as calculated by geNorm, Bestkeeper, and NormFinder**. The samples included: all samples (A), different ages (B), plant tissues (C), cold-treated (D), heat-treated (E), NaCl-treated (F), PEG-treated (G), ABA-treated (H). 1 represents the most stable gene and 10 represents the least stable gene; G: geNorm, B: Bestkeeper, N: NormFinder.

| **Rank** | **Soft-word** | **1** | **2** | **3** | **4** | **5** | **6** | **7** | **8** | **9** | **10** |
| --- | --- | --- | --- | --- | --- | --- | --- | --- | --- | --- | --- |
| **All(A)** | **G** | *UBC* | *aTUB* | *GAPDH* | *UBQ* | *DNAJ* | *ACT7* | *bTUB* | *EF1a* | *SAND* | *CAC* |
|  | **B** | *UBC* | *SAND* | *CAC* | *aTUB* | *EF1a* | *ACT7* | *DNAJ* | *UBQ* | *bTUB* | *GAPDH* |
|  | **N** | *UBC* | *aTUB* | *UBQ* | *GAPDH* | *aTUB* | *ACT7* | *DNAJ* | *SAND* | *EF1a* | *CAC* |
| **Age(B)** | **G** | *aTUB* | *UBC* | *GAPDH* | *bTUB* | *UBQ* | *DNAJ* | *ACT7* | *SAND* | *EF1a* | *CAC* |
|  | **B** | *aTUB* | *UBC* | *bTUB* | *SAND* | *DNAJ* | *ACT7* | *GAPDH* | *CAC* | *EF1a* | *UBQ* |
|  | **N** | *aTUB* | *UBC* | *GAPDH* | *bTUB* | *ACT7* | *SAND* | *UBQ* | *DNAJ* | *EF1a* | *CAC* |
| **Tissue(C)** | **G** | *ACT7* | *DNAJ* | *UBC* | *aTUB* | *SAND* | *UBQ* | *EF1a* | *GAPDH* | *bTUB* | *CAC* |
|  | **B** | *UBC* | *DNAJ* | *ACT7* | *aTUB* | *SAND* | *EF1a* | *CAC* | *bTUB* | *UBQ* | *GAPDH* |
|  | **N** | *DNAJ* | *ACT7* | *aTUB* | *UBC* | *SAND* | *EF1a* | *UBQ* | *GAPDH* | *bTUB* | *CAC* |
| **Cold(D)** | **G** | *SAND* | *ACT7* | *GAPDH* | *UBC* | *DNAJ* | *CAC* | *UBQ* | *aTUB* | *bTUB* | *EF1a* |
|  | **B** | *UBQ* | *aTUB* | *EF1a* | *DNAJ* | *UBC* | *ACT7* | *SAND* | *GAPDH* | *bTUB* | *CAC* |
|  | **N** | *UBC* | *ACT7* | *SAND* | *DNAJ* | *GAPDH* | *UBQ* | *CAC* | *aTUB* | *bTUB* | *EF1a* |
| **Heat(E)** | **G** | *aTUB* | *UBC* | *UBQ* | *SAND* | *GAPDH* | *ACT7* | *DNAJ* | *bTUB* | *CAC* | *EF1a* |
|  | **B** | *aTUB* | *UBC* | *UBQ* | *SAND* | *GAPDH* | *ACT7* | *EF1a* | *CAC* | *DNAJ* | *βTUB* |
|  | **N** | *aTUB* | *UBC* | *UBQ* | *SAND* | *ACT7* | *GAPDH* | *CAC* | *DNAJ* | *βTUB* | *EF1a* |
| **NaCl(F)** | **G** | *DNAJ* | *UBQ* | *bTUB* | *GAPDH* | *UBC* | *SAND* | *aTUB* | *ACT7* | *EF1a* | *CAC* |
|  | **B** | *GAPDH* | *aTUB* | *UBC* | *DNAJ* | *SAND* | *bTUB* | *UBQ* | *EF1a* | *ACT7* | *CAC* |
|  | **N** | *UBC* | *GAPDH* | *SAND* | *aTUB* | *DNAJ* | *UBQ* | *bTUB* | *ACT7* | *EF1a* | *CAC* |
| **PEG(G)** | **G** | *aTUB* | *EF1a* | *bTUB* | *DNAJ* | *UBC* | *CAC* | *UBQ* | *GAPDH* | *SAND* | *ACT7* |
|  | **B** | *DNAJ* | *UBC* | *SAND* | *bTUB* | *EF1a* | *aTUB* | *CAC* | *ACT7* | *UBQ* | *GAPDH* |
|  | **N** | *bTUB* | *aTUB* | *DNAJ* | *UBC* | *EF1a* | *CAC* | *UBQ* | *GAPDH* | *SAND* | *ACT7* |
| **ABA(H)** | **G** | *UBC* | *UBQ* | *bTUB* | *DNAJ* | *SAND* | *ACT7* | *CAC* | *GAPDH* | *aTUB* | *EF1a* |
|  | **B** | *SAND* | *ACT7* | *UBC* | *aTUB* | *DNAJ* | *bTUB* | *UBQ* | *CAC* | *GAPDH* | *EF1a* |
|  | **N** | *UBC* | *UBQ* | *SAND* | *CAC* | *DNAJ* | *ACT7* | *bTUB* | *GAPDH* | *aTUB* | *EF1a* |
